# Identifying Shifts in Leaf-Litter Ant Assemblages (Hymenoptera: Formicidae) across Ecosystem Boundaries Using Multiple Sampling Methods

**DOI:** 10.1371/journal.pone.0134502

**Published:** 2015-07-30

**Authors:** Michal Wiezik, Marek Svitok, Adela Wieziková, Martin Dovčiak

**Affiliations:** 1 Department of Applied Ecology, Faculty of Ecology and Environmental Science, Technical University in Zvolen, Zvolen, Slovakia; 2 Department of Biology and General Ecology, Faculty of Ecology and Environmental Science, Technical University in Zvolen, Zvolen, Slovakia; 3 Department of Aquatic Ecology, Centre of Ecology, Evolution and Biogeochemistry, Eawag Swiss Federal Institute of Aquatic Science and Technology, Kastanienbaum, Switzerland; 4 Department of Environmental and Forest Biology, College of Environmental Science and Forestry, State University of New York, Syracuse, New York, United States of America; Landcare Research, NEW ZEALAND

## Abstract

Global or regional environmental changes in climate or land use have been increasingly implied in shifts in boundaries (ecotones) between adjacent ecosystems such as beech or oak-dominated forests and forest-steppe ecotones that frequently co-occur near the southern range limits of deciduous forest biome in Europe. Yet, our ability to detect changes in biological communities across these ecosystems, or to understand their environmental drivers, can be hampered when different sampling methods are required to characterize biological communities of the adjacent but ecologically different ecosystems. Ants (Hymenoptera: Formicidae) have been shown to be particularly sensitive to changes in temperature and vegetation and they require different sampling methods in closed vs. open habitats. We compared ant assemblages of closed-forests (beech- or oak-dominated) and open forest-steppe habitats in southwestern Carpathians using methods for closed-forest (litter sifting) and open habitats (pitfall trapping), and developed an integrated sampling approach to characterize changes in ant assemblages across these adjacent ecosystems. Using both methods, we collected 5,328 individual ant workers from 28 species. Neither method represented ant communities completely, but pitfall trapping accounted for more species (24) than litter sifting (16). Although pitfall trapping characterized differences in species richness and composition among the ecosystems better, with beech forest being most species poor and ecotone most species rich, litter sifting was more successful in identifying characteristic litter-dwelling species in oak-dominated forest. The integrated sampling approach using both methods yielded more accurate characterization of species richness and composition, and particularly so in species-rich forest-steppe habitat where the combined sample identified significantly higher number of species compared to either of the two methods on their own. Thus, an integrated sampling approach should be used to fully characterize changes in ant assemblages across ecosystem boundaries, or with vegetation change over time, and particularly so in species-rich habitats such as forest-steppe ecotones.

## Introduction

Ants (Hymenoptera: Formicidae) are a varied, abundant, and ubiquitous group of insects that can exert a strong influence as ecosystem engineers on physical, chemical, and biotic properties of terrestrial ecosystems, and especially on soil environment [1]. Ants belong among the most studied terrestrial invertebrates; they have been used to monitor ecosystem health and functioning [2–4], effects of changing climate [5, 6], as well as the effects of changing land-use and successional processes [7, 8].

Our understanding of the role of ants in ecosystems hinges to a large extent on the sampling methodology used to quantify the composition, richness, and abundance (or activity) of ant assemblages. While numerous sampling techniques are available, their suitability can vary substantially across different habitats and ant assemblages [9]. For example, pitfall trap method is the main method used for sampling epigaeic ant assemblages in relatively open habitats such as grasslands, deserts, or shrublands [8,10–14], while leaf-litter sampling method is preferred in forest habitats [15, 16]. Habitat specificity of sampling methods affects our ability to discern patterns present in ant assemblages across ecosystem boundaries (ecotones) and to compare ant assemblages between different ecosystems or within ecosystems undergoing successional changes in vegetation over time (e.g., due to changing climate or land-use). Studies contrasting sampling methods and developing a unified sampling framework that could be used among different ecosystems are needed, particularly in temperate forests and forest-steppe ecotones [17] where such work has been less frequent compared to tropical systems [18–21].

Pitfall trapping involves placement of an open container with a liquid preservative in the ground to trap ants active on the ground surface [9], but they are believed to under-sample litter-dwelling ants [9, 22]. Pitfall traps are cheap, easy-to-install, self-operating, and they can be used for relatively longer time periods [23]. Their main limitations are that samples (capture rates) vary with the locomotion type of ant species [24] and with the physical structure of the ground surface (i.e., thick and compact leaf-litter layer or stones may reduce capture rates; [25]). In addition, preserving agents in the traps may attract some and repel other ant species [23, 26] and catch efficiency can vary with the concentration of the preservative [27].

Leaf-litter sifting techniques have been developed to provide accurate estimates of litter-dwelling ants by measuring their abundance within the sampled volume of leaf-litter [9]. The collected leaf litter is sieved manually or mechanically in an extraction apparatus (e.g., the Winkler extractor, Berlese funnel) in order to extract the ants [9, 23]. Sampling may underestimate abundance during very dry periods (when ants tend to move deeper in the soil; [9]) and for fast moving species (which may be harder to capture; [28]). Relative to pitfall traps, litter sampling is more time consuming, more costly, and it requires destructive sampling of litter [17, 29].

We compared how the two main sampling methods used in characterizing ant assemblages (pitfall traps and leaf-litter sifting) vary in their ability to characterize species richness and community composition across three adjacent temperate ecosystems (relatively mesic beech forest, dry oak forest, and dry forest-steppe ecotone). Accurate quantification of species richness (biodiversity) and community composition are important for our understanding of emergent community and ecosystem characteristics such as ecosystem productivity or compositional stability (e.g., [30, 31]). In Central Europe, beech forests are among the most species-poor forest habitats, usually hosting only few ant species with very low population densities [32], while oak forests and forest-steppe habitats are usually among the most diverse [33, 34]. All three habitat types represent vegetation characteristic of low elevations in Western Carpathians [35] and other areas in Europe [36]. Climate change is likely to alter composition and boundaries of adjacent ecosystem types controlled by moisture and temperature gradients [37–39] and ants may have a potential to serve as early bioindicators of these substantial changes in vegetation structure [2–9, 40], but see also [41]. Ants of warm and relatively mesic forests and forest-steppe habitats are believed to be particularly susceptible to climate warming [5]. The objectives of our study were (*i*) to contrast the ability of pitfall traps and leaf-litter sifting to characterize species composition, richness, and abundance of ant assemblages in adjacent mesic beech forest, dry oak forest, and dry forest-steppe ecotone, and (*ii*) to test whether the combination of the two methods is necessary to provide the full description of the ant assemblages across these adjacent ecosystems.

## Material and Methods

### Study Area

The study was carried out in the Boky Nature Reserve (48° 34´ N, 19° 02´ E, 280–579 m above sea level) located on southern slopes of the Kremnické vrchy Mts. in western Carpathians, Slovakia. The reserve is regarded as one of the most preserved oak dominated old-growth forest in Slovakia, with minimal historical human use [42]. The region has mean annual temperature of 7°C and annual precipitation of 720 mm. Rich brown soils on andesite bedrock and rankers are the dominant soil types. The forests in this area are composed of broad-leaved trees, mainly sessile oak (*Quercus petraea*), Turkey oak (*Q*. *cerris*), European hornbeam (*Carpinus betulus*), and European beech (*Fagus sylvatica*), together with admixtures of maple (*Acer* spp.), lime (*Tilia* spp.) and elm (*Ulmus* spp.). Patches of steppe and forest-steppe vegetation of the class *Festuco-Brometea* typically occur on steep slopes with shallow soils. European beech dominates the forest stands locally in places with increased soil moisture (mainly concave parts of slopes), while oaks dominate the majority of the area and especially exposed slopes and dry ridge tops [43]. The forest stands are characterized by their high stem density (> 850 stems ha^-1^), moderate above-ground growing stock (280 m^3^ha^-1^), and high diversity and amount of deadwood [42]. The reserve has been strictly protected since 1964. We conducted our research based on the research permission No. 2006/00924-Pe issued by the Regional Bureau of Environment in Banská Bystrica, Slovakia.

### Sampling design and data collection

Altogether nine 30 × 30 m study plots were established representing three habitat types: beech dominated forest, oak forest, and oak forest-steppe ecotone (three plots per habitat). The minimum distance between the plots representing a particular habitat was 230 m. All plots were separated by qualitatively different habitat. In each plot we intensively sampled ant assemblages using both pitfall trap method and litter sifting, applied in April, May, and July in 2009.

In each plot, we established a transect consisting of three traps spaced at 5 m intervals, following the procedures used for studying epigaeic ant assemblages [44, 45]. The traps consisted of plastic cups with sealing (5 cm in diameter) buried at soil level and filled with 100 ml of 2% formaldehyde solution with addition of detergent to lower the surface tension. Each trap was left in place for 5 days before being opened in order to reduce digging-in effects [46]. The traps remained open for 5 consecutive days during each month, concluding in 135 trap-days (across 27 traps) per habitat in total.

Leaf litter was sampled at each plot from 16 randomly placed subplots (25 × 25 cm large), with 144 subplots sampled in total across the plots (3) and sampling times (3) per habitat (or the total area of 9 m^2^ per habitat). Most of the surface litter was collected, sieved through the Winkler extractor in order to exclude large particles, and placed into a large polythene bag. Litter-dwelling ants were extracted from the litter using the dry sieve method [47] with a set of three sieves of 12 mm, 5 mm, and 1 mm mesh sizes. From the sifted material the ants were manually extracted and preserved in 60% ethanol solution. Ants were identified at the species level according to [32]; only ant workers were considered in the statistical analyses.

### Statistical analyses

A two factor, partly nested (split-plot) design [48] was used to address our objectives. Total species richness, epigaeic activity (from pitfall traps), density (from sifting), and species composition expressed as qualitative species matrix (based on species presence/absence) as well as quantitative species matrix (based on species activity or density) were used as response variables. For each plot, obtained data were pooled across samples and months to prevent spatial and temporal dependencies and to generalize the responses over the course of the whole sampling period (i.e., May, June, and July). Response variables were compared among the three studied habitat types and two sampling techniques as follows.

First, data from pitfall and sifting samples were analyzed separately using a series of simple linear models. The effect of different habitat types on univariate characteristics (richness, activity, and density) was assessed by one-way analysis of variance (ANOVA). Significant (α = 0.05) results of the overall tests were followed by pair-wise comparisons using Tukey’s HSD tests. Assumptions of each model were screened in diagnostic plots and data were log transformed when necessary. However, we use untransformed data in figures to facilitate interpretation.

Multivariate data (qualitative and quantitative species matrices) were analyzed fitting the same linear models through permutational multivariate analysis of variance (PERMANOVA; [49]). The analyses of those matrices were based on Sørensen [50] and Bray-Curtis [51] dissimilarity, respectively. We included all ant species in the analyses as the preliminary screening showed minimal impact of the rare species on both statistical testing (identical conclusions) and ordination configurations (all Procrustes correlations > 0.95). However, quantitative species data were log-transformed to reduce the influence of dominant species and stabilize the variance. Probabilities of obtained test statistics were based on 9999 unrestricted permutations of raw data [52]. Significant results were followed by relevant pair-wise comparisons. Due to the limited number of permutable units, p values for the pair-wise tests were based on a random Monte Carlo samples from the asymptotic distribution of the test statistics under permutations [53]. P values in pair-wise tests were adjusted by Bonferroni correction to control for family-wise error rate.

Species typical for samples from particular habitat or sampling technique were identified using indicator species analysis [54]. The indicator species analysis was conducted separately for ant activity, density and presence/absence data. The species with high indicator value (IndVal > 70%) were regarded as characteristic species.

Response variables that are directly comparable between sampling methods (richness, qualitative species matrix) were analyzed using partly nested models, with habitat type as a between plot factor (fixed effect), sampling method as a within plot factor (fixed effect), and plot as a factor nested within habitat (random effect). Factor of sampling method consisted of three levels: pitfalls, sifting, and combination of these two methods representing an optimal sampling solution or standard. Species richness was assessed using traditional MANOVA accompanied with Tukey's HSD tests. Species presence/absence matrix was converted to Sørensen dissimilarities and analyzed using PERMANOVA with tests based on 9999 permutations of correct exchangeable units [55]. Again, after the overall differences were found, relevant pair-wise test were adjusted by Bonferroni correction.

In order to facilitate interpretation of the univariate analyses, mean values were plotted along with 95% confidence intervals. Confidence limits were computed by non-parametric bootstrap (10,000 samples) using a bias-corrected accelerated percentile method [56]. Confidence intervals for within plot factor (sampling method) were adjusted for repeated measurements of the same plots [57]. Non-metric multidimensional scaling (NMDS) [58] with half-change scaling and principal component rotation was used to visualize differences in community composition. Scores of indicator species were added into ordinations as weighted sums of particular composition matrices.

Statistical analyses were performed in DISTLM v.5 [59] (Anderson 2004) and R [60] using libraries boot [61], car [62], multcomp [63], nlme [64] and vegan [65].

## Results

We collected a total of 5,338 ant workers belonging to 28 species (Table 1). The total number of species recorded using both methods (pitfall traps and litter sifting) was highest at forest-steppe ecotone (24 species), intermediate in oak forest (17 species), and lowest in beech-dominated forest (11 species).

**Table 1:** Total activity and abundance (density) in beech-dominated forest, oak forest, and forest-steppe ecotone.

Habitat	beech		oak		ecotone	
Sampling method	pit	sift	pit	sift	pit	Sift
**Ponerinae**						
*Ponera testacea* Emery, 1895	-	-	-	-	-	2^2^
**Myrmicinae**						
*Myrmica lonae* Finzi, 1926	-	-	-	-	2^1^	-
*M*. *ruginodis* Nylander, 1846	48^3^	41^3^	22^3^	885^3^	-	-
*M*. *sabuleti* Meinert, 1861	1^1^	1^1^	38^2^	4^2^	31^3^	3^2^
*Stenamma debile* (Förster, 1850)	22^3^	4^3^	8^2^	6^1^	-	1^1^
*Aphaenogaster subterranea* (Latreille, 1798)	-	-	11^2^	4^2^	20^3^	12^1^
*Myrmecina graminicola* (Latreille, 1802)	-	-	-	-	3^2^	-
*Solenopsis fugax* (Latreille, 1798)	-	-	-	-	4^2^	-
*Temnothorax crassisspinus* (Karavaiev, 1926)	57^3^	509^3^	89^3^	505^3^	21^3^	27^3^
*T*. *nigriceps* (Mayr, 1855)	-	2^1^	-	-	-	-
*T*. *parvulus* (Schenck, 1852)	1^1^	11^2^	7^1^	65^3^	42^3^	96^3^
*T*. *unifasciatus* (Latreille, 1798)	-	-	-	1^1^	8^2^	17^1^
*Tetramorium caespitum* (Linnaeus, 1758)	-	-	1^1^	-	16^2^	-
*T*. *moravicum* Kratochvíl, 1941	-	-	-	-	12^2^	2^1^
**Dolichoderinae**						
*Dolichoderus quadripunctatus* (Linnaeus, 1771)	-	-	-	-	1^1^	-
*Tapinoma erraticum* (Latreille, 1798)	-	-	-	-	13^3^	-
**Formicinae**						
*Plagiolepis pygmaea* (Latreille, 1798)	1^1^	-	-	-	25^2^	-
*Lasius alienus* (Förster, 1850)	-	9^2^	4^1^	-	85^3^	2^2^
*L*. *brunneus* (Latreille, 1798)	-	2^1^	-	-	-	-
*L*. *citrinus* Emery, 1922	-	-	-	1^1^	-	-
*L*. *emarginatus* (Olivier, 1792)	9^2^	-	608^3^	121^2^	1603^3^	1^1^
*L*. *niger* (Linnaeus, 1758)	-	-	-	-	1^1^	-
*Camponotus ligniperdus* (Latreille, 1802)	-	-	11^3^	1^1^	8^2^	-
*C*. *fallax* (Nylander, 1856)	-	-	1^1^	-	1^1^	-
*C*. *piceus* (Leach, 1825)	-	-	-	-	5^1^	-
*C*. *aetiops* (Latreille, 1798)	-	-	-	-	1^1^	-
*Formica fusca* Linnaeus, 1758	-	-	3^2^	-	20^2^	-
*F*. *gagates* Latreille, 1798	-	1^1^	3^2^	1^1^	123^3^	2^1^
**Total individuals**	143	584	811	1599	2045	165
**Number of species**	7	9	13	11	22	11

Ant activity was sampled by pitfall traps (pit; number of ants captured per 9 traps and 15 days of sampling) and density was sampled by the leaf litter sifting (sift; number of ants sampled from surface of 9 m^2^). The number of plots where each species was present is indicated by superscript representing the number of plots with species present.

### Variation in species richness, epigaeic activity, and abundance by habitat and method

The two sampling methods differed in the numbers of recorded species (total richness) and they varied in their sensitivity in discerning the effects of habitat type. Across all habitats, we recorded 24 species in pitfall traps and 16 species by litter sifting, with the total of 16 species recorded by only one of the two methods (12 species by pitfall traps and 4 species by litter sifting). The two methods yielded similar estimates of the total species richness in the two forest habitats, but they differed greatly in forest-steppe ecotone where we recorded 22 species in pitfall traps but only 11 with litter sifting (Table 1).

**Fig 1:**
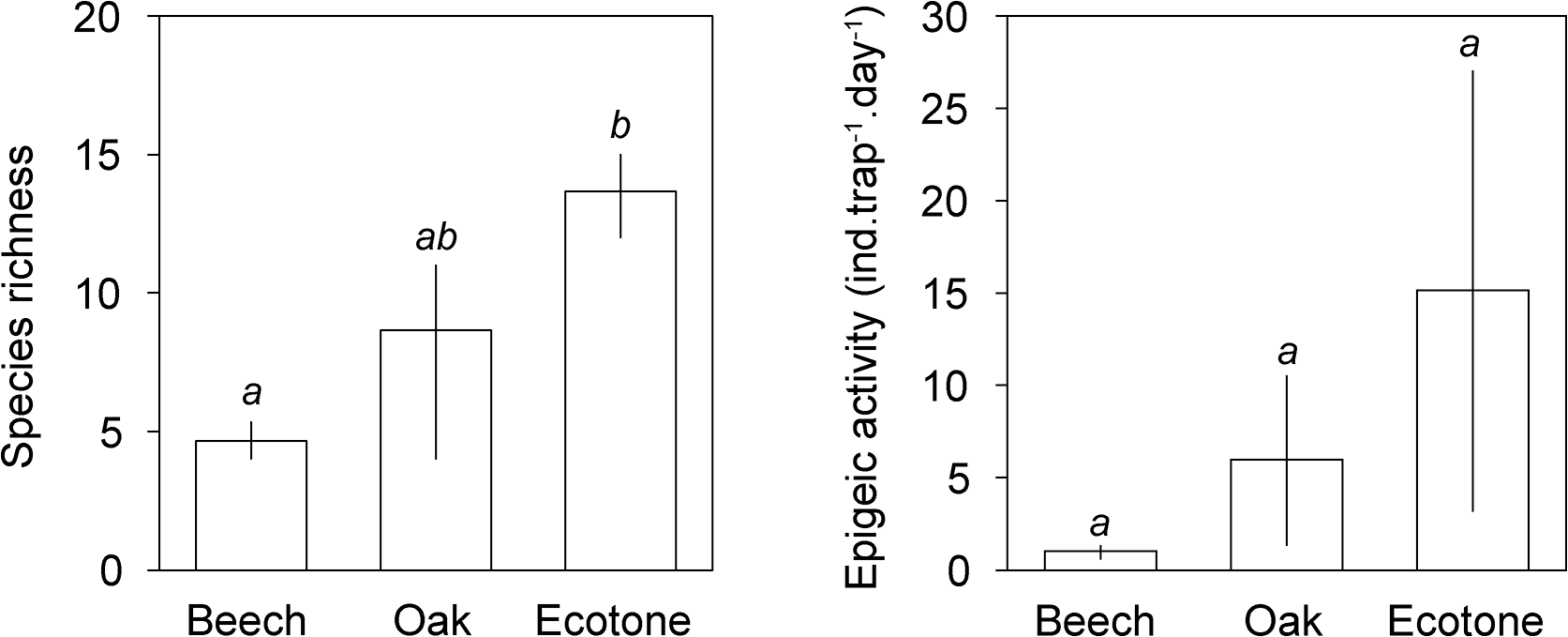
Ant species richness and epigaeic activity (mean ± 95% confidence interval) in beech, oak and ecotone habitats based on pitfall trap collections. Bars with the same lowercase letters are not significantly different.

**Fig 2:**
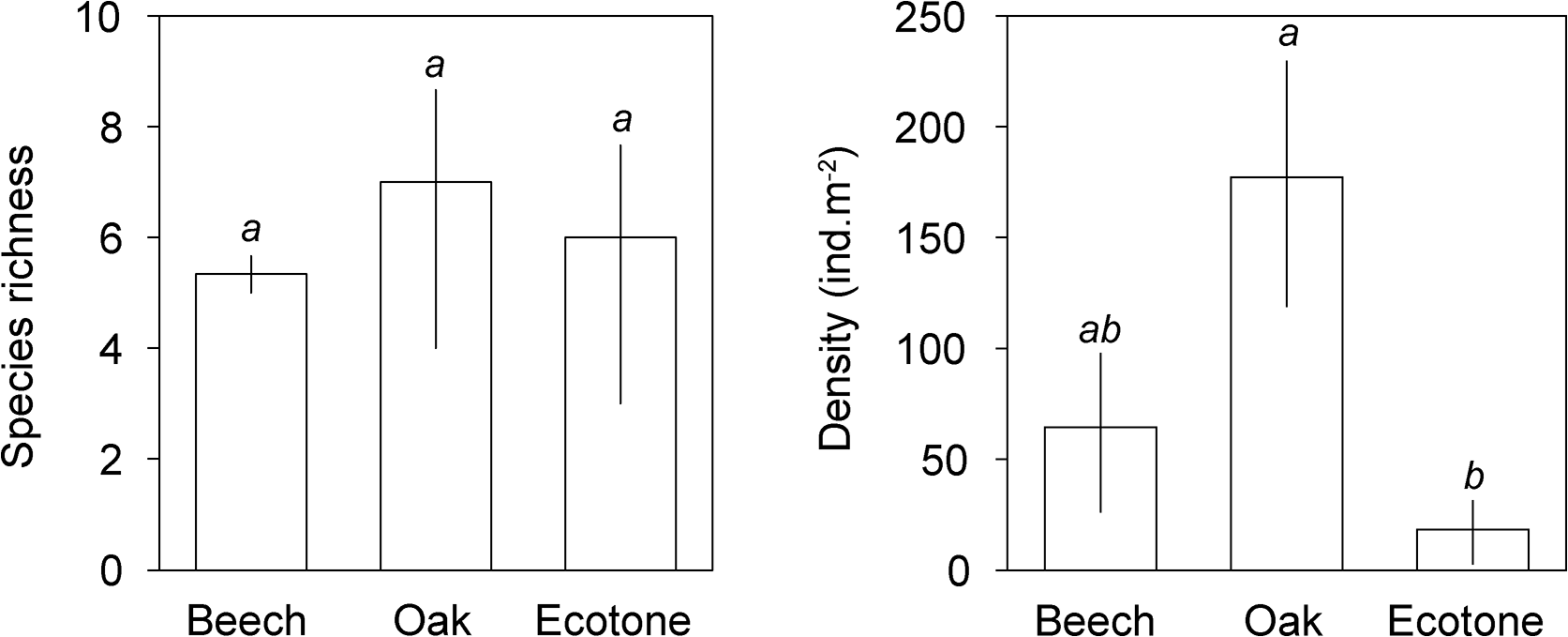
Ant species richness and density (mean ± 95% confidence interval) in beech, oak and ecotone habitats based on sifting method. Bars with the same lowercase letters are not significantly different.

Pitfall trap data revealed a significant effect of habitat on species richness (F_(2,6)_ = 6.35, p = 0.033), but not on overall epigaeic activity (F_(2,6)_ = 2.40, p = 0.172). Forest-steppe ecotone had significantly higher species richness than beech forest, while species richness in oak forest was intermediate and statistically indistinguishable from the other two habitats (Fig 1). In contrast, litter sifting data did not discern significant effects of habitat on ant species richness (F_(2,6)_ = 0.25, p = 0.787), but it suggested significant effects of habitat on ant abundance (F_(2,6)_ = 6.22, p = 0.034). Oak forest had significantly higher abundance of ants than forest-steppe ecotone, while beech forest had an intermediate ant density (Fig 2).

### Variation in species composition by habitat and method

**Fig 3:**
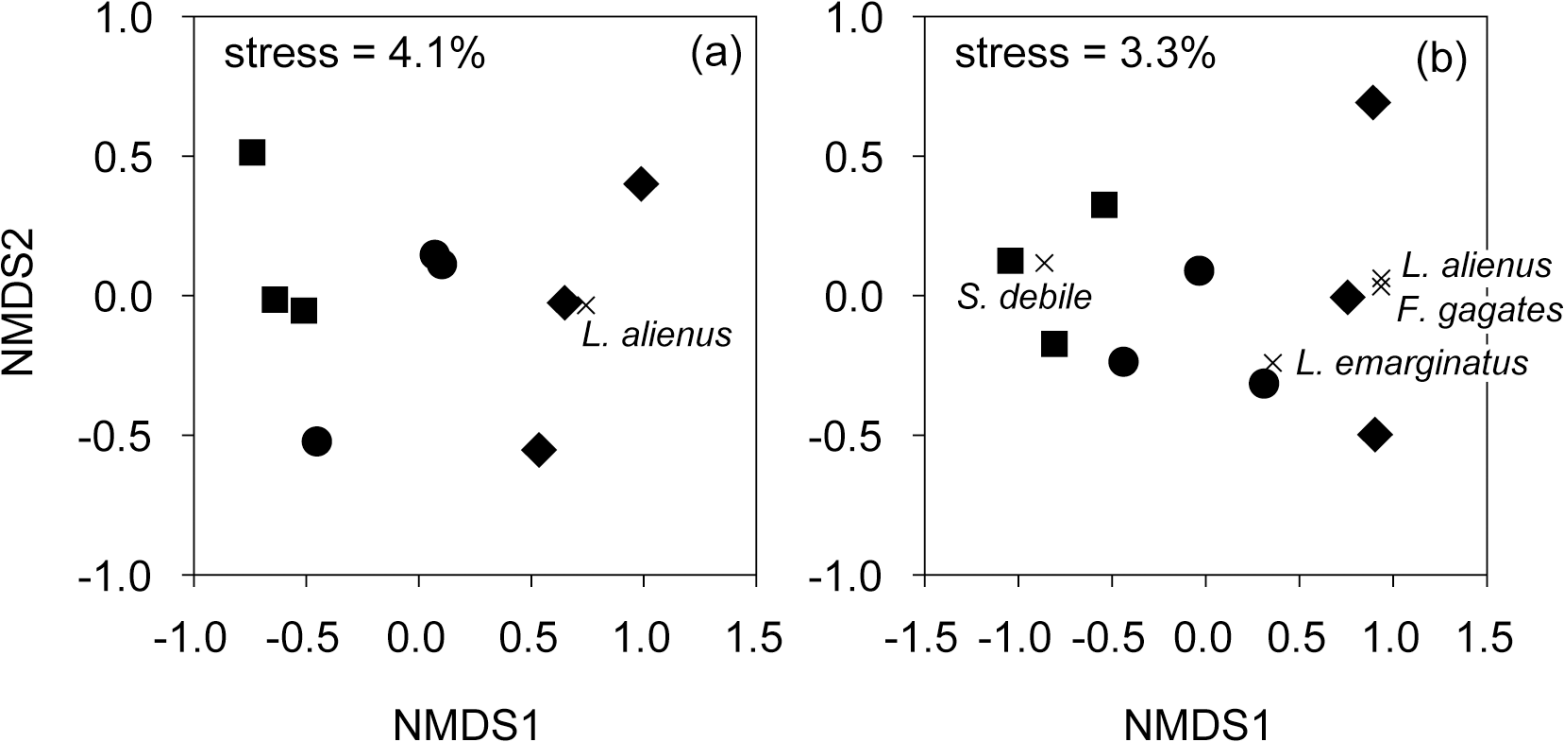
NMDS of pitfall trap data based on qualitative (presence/absence) matrix (a) and quantitative (activity) matrix (b). Squares, circles and diamonds represent beech, oak and ecotone habitats, respectively. Scores of characteristic indicator species (IndVal > 70%) are superimposed as × signs. Values of the final stress are displayed for each ordination.

Community composition detected by pitfall traps showed significant differences between habitats regardless of the type of community matrix used (qualitative: pseudo-F = 5.25, p = 0.010; or quantitative: pseudo-F = 5.35, p = 0.007). Paired comparisons revealed significant differences in species composition between ecotones and beech forests (qualitative matrix: pseudo-t = 3.18, p = 0.031, quantitative matrix: pseudo-t = 3.12, p = 0.007). Characteristic indicator species (IndVal > 70%) were found only for ecotones (*F*. *gagates*, *L*. *emarginatus*, *L*. *alienus*) and beech forests (*S*. *debile*) (Fig 3).

**Fig 4:**
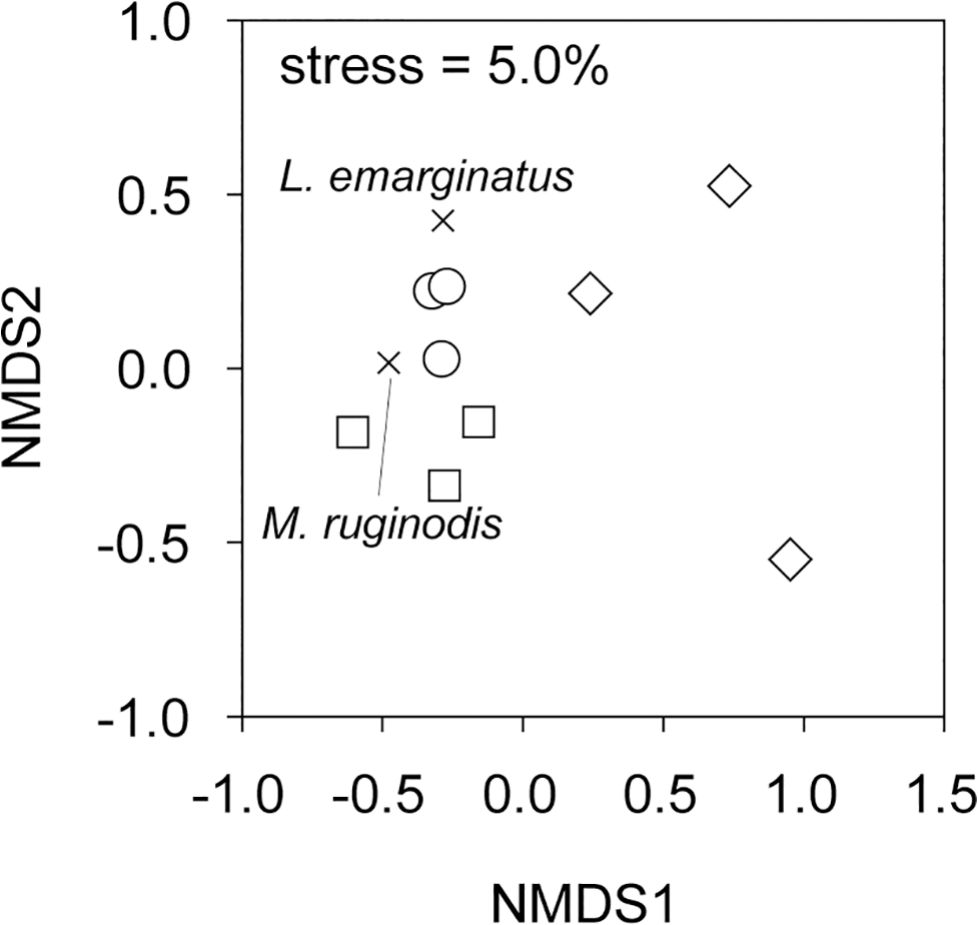
NMDS of sifting method data based on matrix of species density. Squares, circles and diamonds represent beech, oak and ecotone habitats, respectively. Scores of characteristic indicator species (IndVal > 70%) are superimposed as × signs. Value of the final stress is displayed.

Community composition detected by litter sifting also suggested a significant effect of habitat using quantitative community matrix (pseudo-F = 3.56, p = 0.011), but not when qualitative matrix was used (pseudo-F = 1.70, p = 0.169). Nevertheless, due to the low power of tests, we were not able to find significant pair-wise differences among habitats. *L*. *emarginatus* and *M*. *ruginodis* were identified as indicator species for oak forests (Fig 4).

### Quantifying advantages of an integrated sampling approach

**Fig 5:**
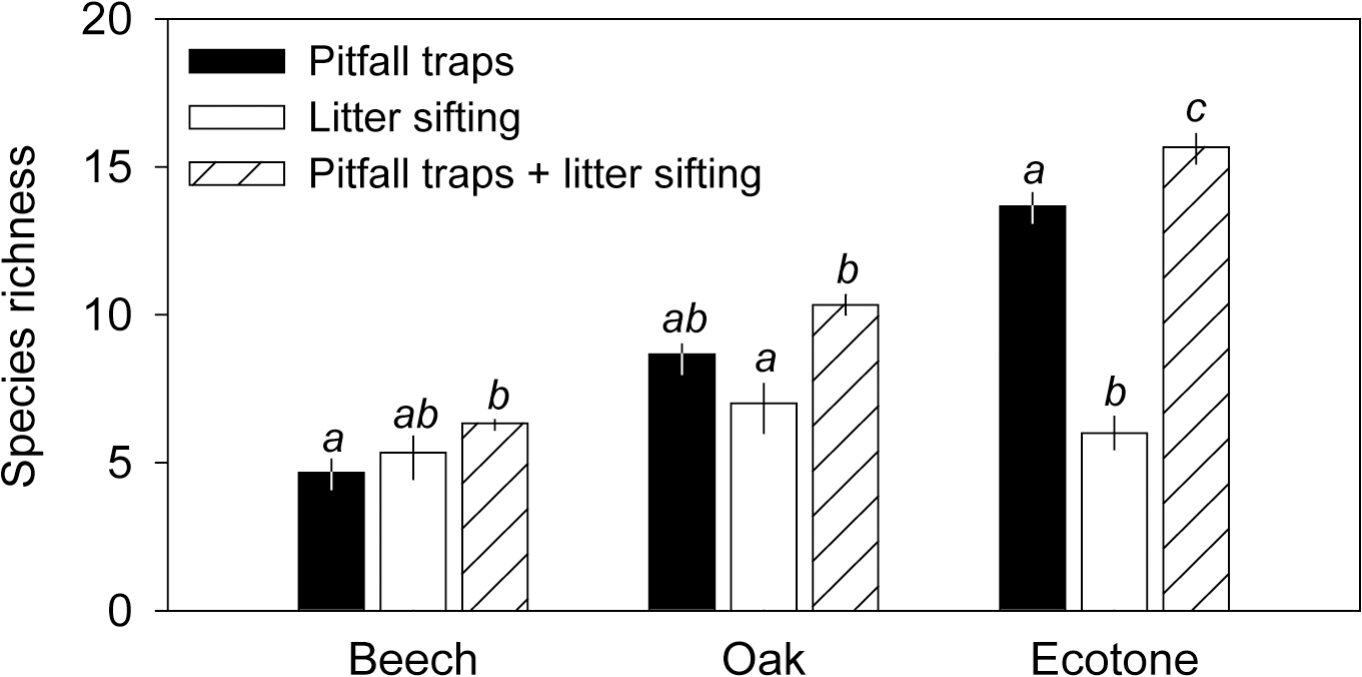
Influence of sampling method on mean ant species richness (± 95% confidence interval) in beech, oak and ecotone habitats. Bars with the same lowercase letters are not significantly different within a particular habitat.

We combined the two sampling methods and contrasted species richness and composition of the combined sample with richness and composition measured by the two methods independently. Split-plot MANOVA on species richness showed that the effect of sampling method depends on habitat (habitat × method: Wilks λ = 0.09, p = 0.010). Significant effect of sampling method was found for all three habitats (Fig 5). The integrated sampling approach yielded better richness estimates than both individual methods, and particularly so in species rich ecotone; the integrated richness estimates were comparable to pitfall trapping in oak forest and to litter sifting in beech forest. Pitfall trapping outperforms litter sifting only in ecotone (Fig 5).

**Fig 6:**
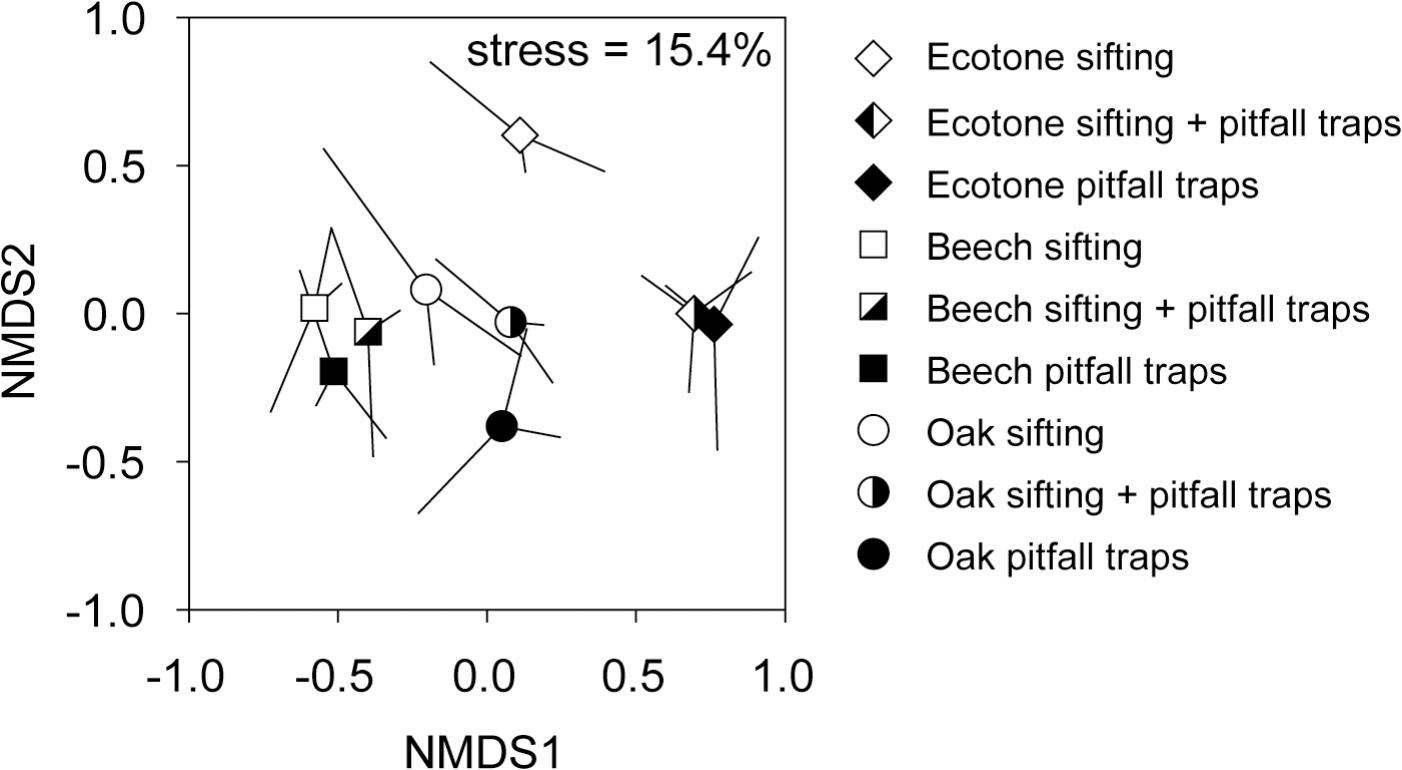
NMDS of pitfall trap data and sifting data based on presence/absence matrix. Geometric shapes (symbols) represent centroids of three independent samples (whiskers). Value of the final stress is displayed.

Multivariate analysis of species composition (using presence/absence data for consistence between the sampling methods) revealed that the sampling methods significantly differed in describing ant assemblages (pseudo-F = 6.86, p = 0.003) independently of habitat type (habitat × method: pseudo-F = 1.21, p = 0.356). The integrated sampling approach and pitfall trap method did not differ statistically in describing community composition (pseudo-t = 3.76, p = 0.117) (Fig 6). On the other hand, community composition revealed by sifting method differed significantly from the integrated sampling approach (pseudo-t = 3.43, p = 0.033). Differences between litter sifting and pitfall trapping were marginally non-significant (pseudo-t = 2.23, p = 0.096).

## Discussion

Our results suggest that the two methods most commonly used in quantifying richness and composition of ant assemblages (i.e., pitfall traps, litter sifting) can lead to considerably different results, and especially so when comparing ant assemblages across different ecosystem or habitat types. The three ecosystem (habitat) types in our study represented a gradient in canopy openness, ranging from the closed forest of shade tolerant beech to a relatively more open oak forest to even more open habitat of the forest-steppe ecotone [43]. Canopy openness strongly influences microclimate [66], one of the main ecological factors shaping ant communities [67–69] and distributions of particular ant species [70]. Ant species richness was found to be negatively associated with canopy tree cover and leaf litter [71], a pattern corresponding well to our overall findings that ant species richness increased from species poor beech forest to species rich forest-steppe ecotone. However, our ability to discern the patterns of species richness across the three habitats rested on the pitfall trap sampling; the sifting method failed to differentiate the three habitats in terms of species richness. The low power of litter sifting to detect the strong richness gradient present across the three ecosystems is not surprising given that the number of ant species associated with forest litter was low compared to the number of epigaeic species sampled by pitfall traps in more species rich habitats. Yet, the inclusion of litter sampling in the integrated sampling approach made species richness estimates more accurate, and especially so in species rich ecotone where the integrated sampling yielded more accurate richness estimates compared to either of the two methods used on their own.

While pitfall traps confirmed the expected patterns in species richness and epigaeic activity (lowest in beech forest and highest in forest-steppe ecotone), litter sifting represented a different picture with the abundance (density) of ants lowest in forest-steppe ecotone and highest in oak forest. Importantly, the sifting method yielded higher numbers of individuals compared to pitfall traps in both forest habitats, and especially so for the dominant litter species (e.g., *Myrmica ruginodis*, *Temnothorax crassispinus*). However, compared to pitfall traps, sifting method appeared ineffective in our study in sampling both (*i*) open habitat specialists (e.g., *Plagiolepis pygmaea*, *Tetramorium caespitum*, *Solenopsis fugax*), which tend to avoid leaf litter [32], and (*ii*) fast moving forest and forest-steppe species that evaded litter extraction [28] but were characterized as frequent across all three habitats based on our pitfall trap data (e.g., *Lasius emarginatus*). Although our results corroborate previous findings that pitfall trap method is appropriate for open habitats and litter sifting method for forest habitats [9, 10, 22, 72], they also suggest that the pitfall trap method worked well also in forest ecosystems. Overall, the combination of the two methods was beneficial for characterizing species richness across both specialist groups (leaf-litter *vs*. open habitat) and locomotion types (fast *vs*. slow), and particularly so in species rich ecotones where neither method on its own fully captured all species [17].

Our work improves the current understanding of ant assemblages and sampling methods in beech and oak forests and forest-steppe transitions in Europe. Although little has been published on the composition of ants assemblages in European temperate broadleaved forests, our results corroborate the few previous studies of litter-dwelling ant assemblages of oak forests in confirming that these assemblages tend to be quite diverse and dominated by a few common forest species (especially *Temnothorax crassispinus*, *Stenamma debile* and *Myrmica ruginodis*) accompanied by a relatively broad array of other less abundant forest ant species [34, 73, 74]. Interestingly, *Lasius emarginatus*, a very abundant species in our study, was very rare or missing in the other published accounts [34, 74]. In addition to observing these characteristic forest species, we identified a diverse set of indicator species for the relatively more open forest-steppe ecotone. This indicator species group well reflected the transitional character of forest-steppe ecotone since it included forest-steppe specialists (*Formica gagates*, [33]), species typical of abandoned grasslands colonized by shrubs (*Lasius alienus*, [8]), as well as forest specialist species (*Lasius emarginatus*, [34]). Overall, the pitfall trap method proved to be appropriate for characterizing compositional shifts among the three habitats in our study. While both litter sifting and pitfall trap method revealed significant compositional differences between the species-poor forest habitat and ecotone, the pitfall trap data were more sensitive and lead to a greater number of indicator species identified in forest-steppe ecotone. On the other hand, the sifting method revealed litter-dwelling indicator species for oak forest (unidentified by pitfall trap method), confirming the strength of the integrated sampling approach.

Quantifying and understanding the differences in ant assemblages across ecosystems is important especially when vegetation character and ecosystem boundaries change over time as they respond to changing climate or land use [8, 75]. Habitat temperature regime can be dramatically affected by vegetation structure (i.e., canopy openness) and temperature influences many aspects of ant biology such as ant functional responses, species range limits, and the diversity of ant assemblages [6, 15, 76]. Ants from lower elevations (with warmer climate and sometimes more open habitat) can be more susceptible to negative effect of climate warming [5]. Forest-steppe ecotones and oak dominated forest in our study represent some of the plant and ant communities most adapted to warm and dry climate within the deciduous forest biome in Europe. With changing climate, these communities are expected to expand over time at the expense of more mesic forest communities such as beech forest [39]. Consequently, robust methods for monitoring these future changes across forest ecosystem boundaries are needed.

In this study, we demonstrated that an integrated sampling framework utilizing different sampling methods can appropriately address distinct aspects of ant communities inhabiting different ecosystems, and thus it can overcome specific disadvantages of using only a single method to monitor ant community changes across ecosystem boundaries. The integrated sampling approach is well known to myrmecologists worldwide [9], however it is not always used in myrmecological studies. We apply it in a novel way to vegetation transition zones that are likely to change as the result of climate or land-use changes [77], and in habitats in which it has not been applied in Europe before. We are not aware of any other research explicitly stating with quantitative support that transition zones need to be sampled using integrated methods due to their dynamic character and due to global environmental changes.

## Supporting Information

S1 Dataset Detailed capture data. (XLS)
